# The role of gene-environment interactions in endocrine-sensitive life stages for shaping mental health: focus on the RE-MEND project

**DOI:** 10.3389/fpsyt.2026.1738584

**Published:** 2026-02-20

**Authors:** Khawla Abualia, Andrea Cediel-Ulloa, Philip Allsopp, Angelika Augustine, Jonas Bergquist, Carl-Gustaf Bornehag, Karin Broberg, Nicolò Caporale, Erika Comasco, Diego di Bernardo, Rosário Domingues, Elina Drakvik, Chris Gennings, Malin Gingnell, Daniel Globisch, Maria Kippler, Emeir McSorley, Maria Mulhern, Hitesh V. Motwani, Ivan Nalvarte, Anna Oudin, Anisur Rahman, Doreen Reifegerste, Theo Rein, Alkistis Skalkidou, J. J. Strain, Giuseppe Testa, Liudmyla Tsiukalo, Edwin van Wijngaarden, Alison Yeates, Joëlle Rüegg, Philipp Antczak

**Affiliations:** 1 Department II of Internal Medicine, Faculty of Medicine and University Hospital Cologne, University of Cologne, Cologne, Germany; 2 Center for Molecular Medicine Cologne (CMMC), University of Cologne, Cologne, Germany; 3 Department of Organismal Biology, Uppsala University, Uppsala, Sweden; 4 Nutrition Innovation Centre for Food and Health, Ulster University, Coleraine, United Kingdom; 5 School of Public Health, Bielefeld University, Bielefeld, Germany; 6 Department of Chemistry for Life Sciences, Uppsala University, Uppsala, Sweden; 7 Department of Health Sciences, Karlstad University, Karlstad, Sweden; 8 Institute of Environmental Medicine, Karlinska Institutet, Stockholm, Sweden; 9 Department of Oncology and Hemato-Oncology, University of Milan, Milan, Italy; 10 Human Technopole, Milan, Italy; 11 Department of Women’s and Children’s Health, Uppsala University, Uppsala, Sweden; 12 Department of Chemical, Materials and Industrial Engineering, University of Naples “Federico II”, Naples, Italy; 13 Telethon Institute of Genetics and Medicine, Naples, Italy; 14 Department of Chemistry, University of Aveiro, Aveiro, Portugal; 15 Research Programs Unit, Faculty of Medicine, University of Helsinki, Helsinki, Finland; 16 Department of Environmental Medicine, Icahn School of Medicine at Mount Sinai, New York, NY, United States; 17 Department of Medical Sciences, Experimental Cognitive and Affective Neuroscience Lab, Uppsala University, Uppsala, Sweden; 18 Science For Life Laboratory, Uppsala University, Uppsala, Sweden; 19 Department of Environmental Science, Stockholm University, Stockholm, Sweden; 20 Department of Neurobiology Care Sciences and Society, Karolinska Institutet, Stockholm, Sweden; 21 Department of Laboratory Medicine, Lund University, Lund, Sweden; 22 Department of Epidemiology and Global Health, Sustainable Health, Umeå University, Umeå, Sweden; 23 Maternal and Child Health Division, International Centre for Diarrhoeal Disease Research, Bangladesh (ICDDR,B), Dhaka, Bangladesh; 24 Department of Translational Research in Psychiatry, Max Planck Institute of Psychiatry, Munich, Germany; 25 The Department of Community and Preventive Medicine, University of Rochester School of Medicine and Dentistry, Rochester, NY, United States; 26 Cologne Excellence Cluster on Cellular Stress Responses in Aging-Associated Diseases (CECAD), University of Cologne and University Hospital Cologne, Cologne, Germany

**Keywords:** endocrine-sensitive life stage, mental health, mental illness, resilience, stigma, susceptibility

## Abstract

The number of people seeking help for mental illness is increasing across all ages, creating a major burden for individuals, families, and the society. While personalized medicine is advancing in other fields, diagnosis and treatment of mental disorders remain largely symptom-based and fail to capture individual, sex, and gender differences in risk, manifestation, and treatment response. Early signs of illness often go unnoticed due to the lack of monitoring tools, and stigma continues to hinder prevention and care. In some phases of life, an individual’s susceptibility to mental illness is heightened and may be influenced by changes in endocrine signalling. To address these challenges, the research project Building REsilience against MEntal illness during ENDocrine-sensitive life stages (RE-MEND) has implemented an interdisciplinary approach focusing on four critical endocrine-sensitive life stages: prenatal, puberty, peripartum, and older age. The project integrates longitudinal population-based cohorts with experimental and clinical studies to identify genetic, environmental, and endocrine factors shaping susceptibility and resilience to mental illness. Multi-omics data (genomics, epigenomics, transcriptomics, proteomics, metabolomics, lipidomics, and adductomics) will be combined with neurobiological, clinical, and behavioural measures, analysed using advanced biostatistics and machine learning. RE-MEND seeks to i) identify risk and resilience factors affecting mental health; ii) deliver biomarker panels for susceptibility, disease progression, and treatment response across sensitive life stages; iii) discover novel drug targets through repurposing strategies, and iv) promote mental health literacy and reduce stigma. The integration of biological research with communication science is anticipated to result in translatable findings, supporting earlier intervention and more effective care.

## Introduction

The World Health Organization (WHO) defines mental health as “a state of mental well-being that reflects the individual’s ability to adapt to the different life situations and be aware of their own needs and responsibilities and be productive in their societies” (1). Studies reported that 15% of individuals worldwide and 18.5% in the European Union (EU), suffer from a mental disorder, with anxiety (4% worldwide, 5.5% EU) and depression (3.8% worldwide, 5% EU) as the most common mental illnesses (2–4). Mental conditions can affect a person’s cognitive, psychomotor, and emotional processes, leading to a broad spectrum of symptoms that disrupt daily life (2). People with mental health symptoms are also at higher risk of poorer physical health, making them more vulnerable to obesity and other chronic conditions, further compounding their challenges (5). In fact, individuals with mental disorders such as schizophrenia, bipolar disorder, and chronic depression with psychotic features have a reduced life expectancy of around 10–20 years compared to the general population, to a large extent due to physical health conditions, inadequate health care access, and the higher suicidal rates (6, 7). Apart from individual suffering, the increased treatment costs and individual’s decreased productivity creates additional pressure on the patient and the community (8).

Different screening tests have been developed, yet these rely largely on verbal reports and questionnaires from the patients, and to a lesser extent on biological assessments (9). To improve diagnosis of common mental disorders, the scientific community has worked extensively to understand the underlying mechanisms that contribute to mental illnesses. Mental disorders display moderate heritability (10, 11), and linkage analysis studies have identified multiple genes that play a crucial role in brain development and that are associated with conditions such as depression (12). Researchers have also investigated other biological factors contributing to the aetiology and the pathophysiology of mental illnesses. These investigations have identified several potential factors including alterations in neurotransmitter signalling, disruptions in the hypothalamic-pituitary-gonadal and hypothalamic-pituitary-adrenal axes as well as changes in immune cytokine levels (13–15). Additionally, the prevalence of common mental disorders, including depression and anxiety, has been noted to be higher in women than in men, which has been partly attributed to the hormonal differences between sexes (16).

The rapid urbanization of societies over the past century has dramatically increased exposure to several environmental and social stressors, which has the potential to modulate both mental and physical health outcomes, especially if encountered during hormonal sensitive life stages (17, 18). Examples are chemical, noise, and light exposures, dietary factors, adverse early life experiences, as well as lack of greenery spaces and social support. However, the interaction between environmental factors and the pathophysiology of mental health disorders is far from fully understood (19–22).

Beyond the challenges in diagnosis and biological understanding, research in health communication science has shown that communication strategies can significantly impact mental health literacy (MHL), which comprises the ability to identify mental health disorders, knowledge about causative and risk factors, treatments, attitudes towards mental health disorders (including stigma), and help-seeking or preventive behaviour (23). However, little is known about the potential effects of health communication considering gene-environment interactions, as communication research has mostly focused on comparing nurture vs. nature framing (24).

Taken together, there is evidence that biological factors play a critical role in both risk and resilience to/for mental health and that they may hold diagnostic utility (25, 26). However, current studies regarding mental disorders lack comprehensive integration that bridges the gap surrounding molecular and neurobiological understanding of mental states. This gap has hindered progress in both diagnosis and treatment of mental illness, with no significant improvement in recent years (27). Many studies have focused on identifying risk factors, while the biological basis of resilience remains understudied, particularly during vulnerable endocrine-sensitive life stages. There are also critical gaps in our understanding of how hormonal changes interact with behavioural outcomes, and how complex biological systems, such as epigenome, proteome, metabolome, lipidome, endocrine signalling pathways, and neural biomarkers, interact with environmental stressors to influence mental health state. The absence of effective tools for early detection and biology-based treatment often results in delayed and less effective therapy, increasing the societal burden of mental illness. “Multi-omics” approaches, assessing several biological systems simultaneously, are promising, yet rarely integrated, and the current biomarker discovery efforts are based on clinical cohorts rather than population-based studies, which limits the identification of early-stage indicators. Finally, there is insufficient mental health literacy and education among health care professionals, intervention developers, and the public, as well as stigmatization of affected individuals. Together, these limitations prevent translation of knowledge into effective implementation of personalized diagnosis and therapy, discovery of novel biomarkers and drug development, and elaboration of comprehensive guidelines for early interventions and prevention.

**Figure 1:**
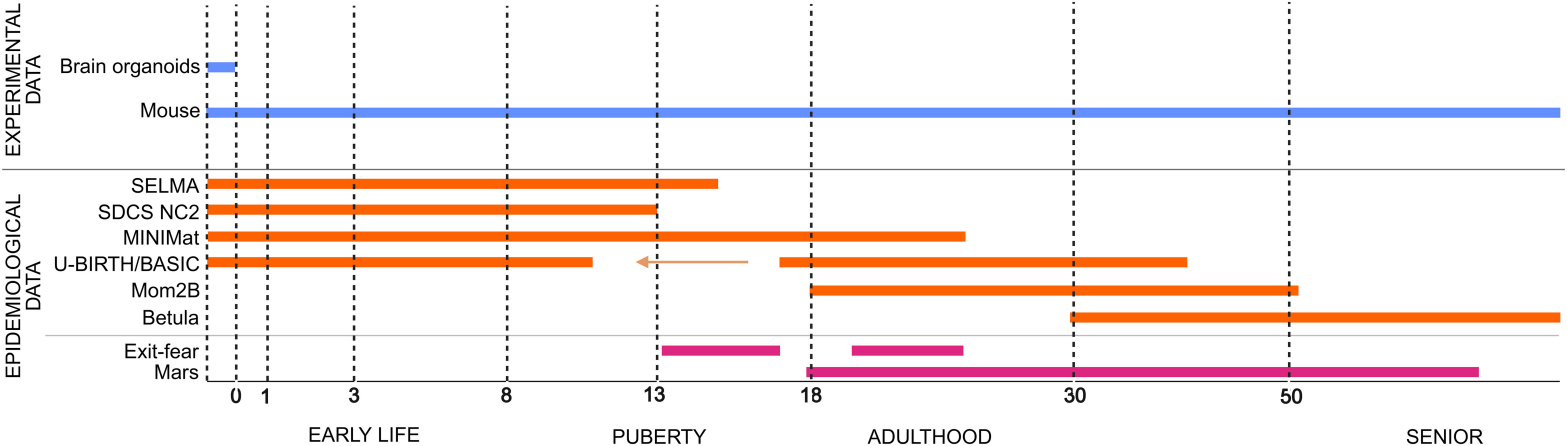
Cohorts and experimental studies across life stages starting from pregnancy and early life within RE-MEND. Epidemiological data are obtained from the six different population-based cohorts (SELMA, SCDS NC2, MINIMat, U-BIRTH/BASIC, MoM2B, and Betula), as well as two clinical studies (EXIT-fear and MARS). Orange lines represent population-based cohorts. Data from BASIC cover pregnancy period and the children (up to 11 years old) from the included pregnant mothers - represented by an arrow. Clinical studies - represented in magenta. Experimental data will be derived from the *in vitro* brain organoids study and the *in vivo* mouse study - represented in blue. Figure created in https://BioRender.com.

To address these gaps through an integrated approach, the EU-funded Building REsilience against MEntal illness during ENDocrine-sensitive life stages (RE-MEND) project was established, which investigates six large population based prospective cohorts, spanning four different life stages that cover susceptible phases for mental illness, to identify environmental and genetic risk and resilience factors that affect the individual’s mental health state. In parallel, experimental studies using brain organoids and a mouse life stage model are employed to validate observational findings, generate mechanistic knowledge, and identify associated biomarkers that contribute to understand how individuals become susceptible or resilient to mental ill-health across sex and age groups (**Figure 1**). Moreover, the project will use these discoveries to identify potential novel drug targets through drug repurposing combined with proof-of-concept experimental studies. These results will then be communicated to the public through evidence-based communication approaches and the establishment of educational resources surrounding mental health. This enables RE-MEND to establish links between genetics and environmental factors that define mental health states in the general populations before clinical signs are states are reached. It further makes the underexplored connection between programming for vulnerability and resilience as well as triggering of mental ill-health and endocrine signalling, and thus also biological sex, across different life stages.

This manuscript provides an overview of the planned activities within RE-MEND coupled to the current state of knowledge at the life stages explored in the project.

## Interaction between environmental factors and genetics in endocrine-sensitive life stages

**Figure 2:**
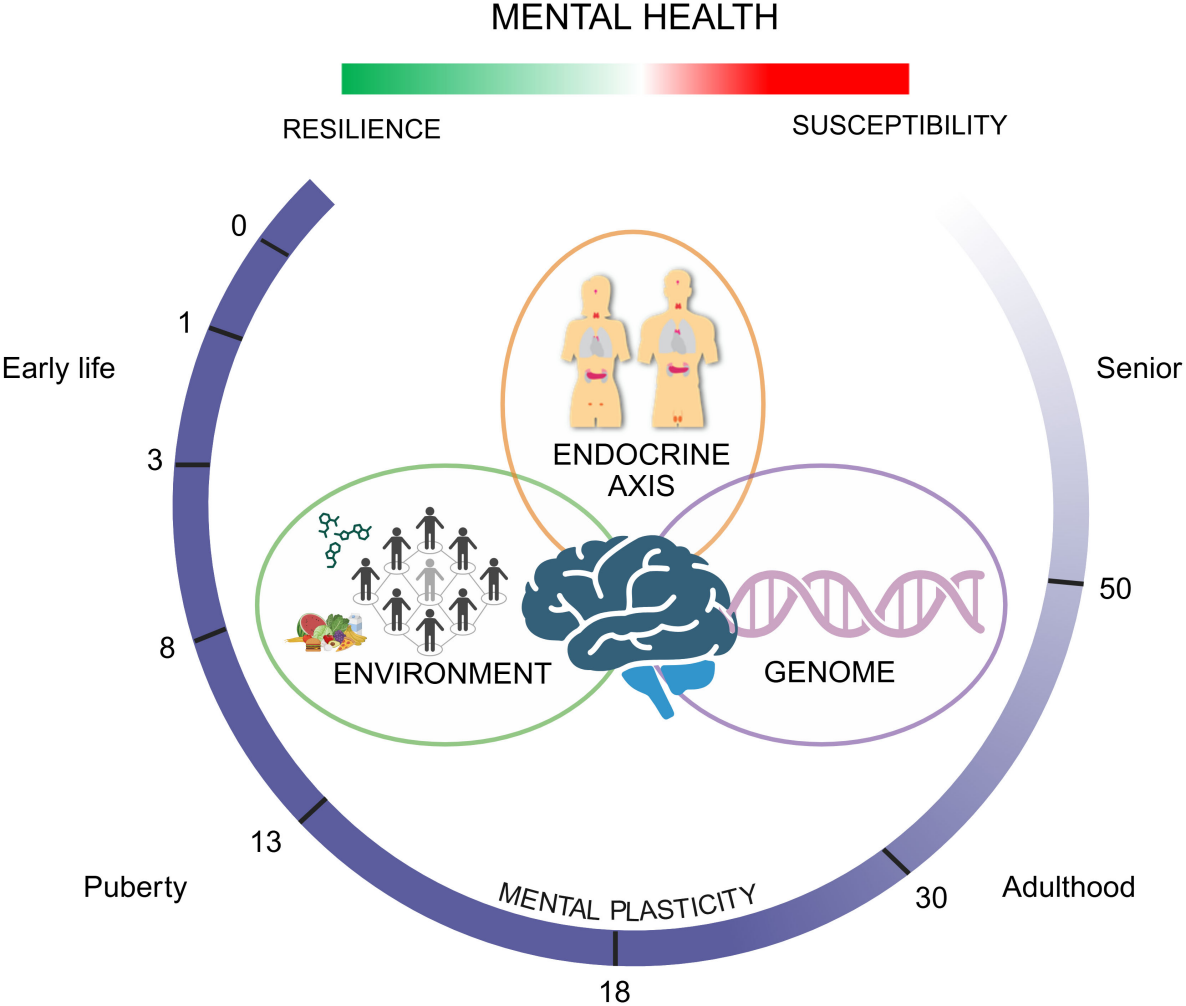
Overview of the RE-MEND framework. RE-MEND explores how mental health is influenced by the interaction of genetic and environmental factors across four key endocrine-sensitive life stages: early development, adolescence, the peripartum period, and later adulthood. These critical periods involve major hormonal shifts that may increase vulnerability or promote resilience to mental illness. The project aims to uncover how these interacting factors shape individual susceptibility or resilience to psychiatric conditions across the lifespan. Figure created in https://BioRender.com.

The concept underlying RE-MEND is that, throughout lifetime, the interplay between the environment and the genome shapes an individual’s mental state. At critical phases in life, an individual’s susceptibility to mental illness is heightened; these “programming phases” are most prominent during early life (i.e., prenatal period and early childhood), and re-occur during challenging phases later on in life, such as puberty, pregnancy, and transition into old age. Notably, such programming and challenging phases are at least partly driven by endocrine stimuli and often characterised by large hormonal fluctuations, which implicates an important role of the endocrine system in shaping and controlling mental health. RE-MEND focuses on critical life phases where endocrine signalling is crucial for shaping an individual’s current or future mental state: i) during pre- and early post-natal neurodevelopment; ii) during puberty; iii) peripartum; and iv) during the transition to older age, including menopause (**Figure 2**).

### Early life

The period spanning the third gestational week to early adulthood marks a particularly vulnerable phase of human brain development. During this prolonged developmental period, the brain is highly susceptible to environmental factors influencing its maturation process, especially in the early stages when the protective blood brain barrier (BBB) and other defence mechanisms are not yet fully developed (28).

Several environmental risk factors have been reported to be associated with mental health later in life, including maternal factors and placental dysfunction that can comprise foetal brain development (29). In addition, chemical exposures, particularly to endocrine-disrupting compounds (EDCs) and neurotoxic metals, represent critical environmental stressors (30). EDCs are exogenous substances that interfere with the hormonal system and thereby induce adverse health outcomes. EDCs, such as bisphenols, phthalates, per- and polyfluoroalkyl substances (PFAS), are commonly used industrial chemicals and pesticides, and found, e.g., in plastics, food packaging, and personal care products (31). Increasing evidence links early life exposure to EDCs to impaired brain development (32) and neurodevelopmental outcomes such as behavioural difficulties, hyperactivity, aggression, anxiety, depression, and inattention (33). Neurotoxic metals such as methylmercury and lead, commonly found in food, consumer products, and water supplies, can damage neurons and interfere with essential processes for brain development (34–36). Moreover, early psychosocial stressors, such as adverse life events (ALE) during pregnancy period or adverse childhood experiences, interfere with socioemotional development in children (37) and lead to changes in the brain structure and function (38). The severity of such stressors correlates with the likelihood for exposed children to develop depression in adulthood (39).

The exact mechanism of how early environmental stress contributes to mental illness is not fully understood. However, several studies support a role of stress in the hyperactivation of key neuroendocrine systems, such as the hypothalamus-pituitary-adrenal (HPA) axis, which can persist during adulthood (40). Furthermore, early stressful experiences, such as maternal separation, have been suggested to increase the secretion of brain-derived neurotrophic factor (BDNF) in the central nervous system (41), and to elevate the levels of serotonin and dopamine in the brain (42, 43). Different family studies have been conducted to identify susceptibility genes underlying psychiatric disorders in children (44). These studies demonstrated that familial risk varies across different conditions, with children of affected parents showing increased risk for mental disorders such as depression compared to the general population (45).

In addition, recent studies have shown that a number of genetic variants are associated with childhood psychiatry disorders. For example, the Dopamine Receptor D4 gene (*DRD4*) has been consistently associated with Attention Deficit Hyperactivity Disorder (ADHD) (46). Current research efforts are concentrated on identifying further genetic variants that increase the risk of mental disorders (47). However, the presence of polygenic risk score tools, that generate a numerical value estimating the individual’s genetic susceptibility to a trait or disorder based on the combined effects of many genetic variants, are not yet sufficient to provide valuable clinical information in the field of mental illness (48).

The epigenome is often seen as the molecular bridge between genetic and environmental effects. The epigenome, heritable but reversible biochemical modifications on or around the DNA that alter its structure but not its sequence, plays an important role in the development of the central nervous system. Epigenetic modifications occur through mechanisms such as DNA methylation, histone modifications, and non-coding RNA regulation, and influence various receptors and hormones involved in brain function (49). Early environmental stressors can induce epigenetic alterations, including DNA methylation changes in stress-related genes like the glucocorticoid receptor Nuclear Receptor Subfamily 3, Group C, Member1 (*NR3C1*), and the *BDNF* gene, thereby increasing susceptibility to mental disorders such as depression and anxiety later in life (50).

RE-MEND addresses the hypothesis that environmental exposures (such as EDCs, stress, diet/nutrition) interfering with endocrine signalling involved in brain development, in interaction with an individual’s genome, program mental health susceptibility and resilience. To examine the interaction between genetics, early life environmental exposure, and mental health in children, two longitudinal cohort studies will be used within RE-MEND: the Swedish Environmental Longitudinal, Mother and child, Asthma and allergy cohort (SELMA) (51), and the Seychelles Child Development Study Nutrition Cohort 2 (SCDS NC2) (52). SELMA is a Swedish pregnancy cohort study in which 2300 women were recruited during their first trimester of pregnancy, and their children have been followed up until puberty. At median gestational week 10, exposure to 26 different chemicals with suspected or known endocrine-disrupting properties was assessed. Furthermore, data were collected regarding mothers’ IQ, nutrition, adverse life events during pregnancy, and potential confounders. For the children, extensive health data were collected and are available at different ages. In addition, within RE-MEND, behavioural tests (Strength and Difficulties Questionnaire (SDQ), Social Responsiveness Scale (SRS) together with psychological tests at age 15), along with genomic and epigenomic data at ages 7 and 15 will be used. The second study, SCDS NC2, includes 1,522 mother-child pairs with data on child behaviour at age 7, as well as different biomarkers of nutrition, dietary pattern, genomic and epigenomic data and methylmercury levels in both the mother and their children. Genomic data will be used to construct polygenic risk scores and investigate their contribution to mental health outcomes without and in combination with environmental factors. As it is hypothesised that epigenetic changes could mediate exposure-associated mental health outcomes, epigenomic data will be used in formal mediation analyses. Additionally, epigenomic patterns strongly associating with exposure and/or outcomes will be further assessed for their validity as biomarkers. Given that these two cohorts originate from genetically distinct populations with different exposure profiles, their data will be used individually to identify factors that, in interaction with the genome, increase or decrease the risk for mental health problems and to uncover molecular signatures that mediate these interactions. Commonalities between the cohorts will be explored and evaluated through the use of risk scoring profiles linking the behavioural tests and molecular responses observed in both cohorts.

### Puberty

Another critical period of rapid hormonal changes and for crucial neurodevelopmental processes is puberty. This stage is highly sensitive due to physiological and psychological changes, as well as a steep increase in societal demands related to self-assertiveness and social relationships. This turmoil can increase vulnerability to mental health issues, with a clear increase in the prevalence of psychiatric symptoms within this age group. Girls, in particular, are nearly twice as likely as boys to experience major depression (53). The peak age for puberty-related mental health issues, including anxiety disorders, panic, and posttraumatic stress disorders, is between 14.5 and 18 years (54). These conditions carry a high risk of recurrence later in life (55).

The emergence of mental health issues during puberty remains poorly understood but involves complex interactions between environmental, sociological, and biological factors, including genetic predisposition and epigenetic modifications (56, 57). Environmental and sociological risk factors, such as early life trauma, stressful family environment, peer relationship difficulties, and academic pressure may contribute to early puberty onset and exacerbate adolescent mental health issues (58–61). Especially early life trauma has been shown to be related to early entrance into puberty which is a long-known risk factor for the development of mental health problems later in life (62).

Genetic factors also play a role in increasing vulnerability to mental health issues during adolescence with variants in genes such as Catechol-O-Methyltransferase (*COMT*), or the serotonin transporter linked polymorphic region gene (*SLC6A4* also called *5-HTTLPR*), and *BDNF* potentially increasing susceptibility to develop mood and anxiety disorders during pubertal transition (63–65).

During puberty, gonadal steroid levels rise in both sexes. Since receptors for these hormones are widely spread in the brain, pubertal changes can significantly affect brain structure, function, and connectivity (66). These hormonal influences interact with genetic and environmental factors, creating individual-specific risk profiles (67). Some hormonal changes can also induce DNA methylation and histone modifications in stress-response genes, altering their expression patterns (68).

Adolescents enter puberty with different neurobiological and psychosocial predispositions that are shaped by genetics and epigenetics, environmental and sociological influences (57, 69). However, research examining the impact of these interactions on mental health during puberty remains limited, particularly in females (53), highlighting the need for more comprehensive and multilevel studies.

Based on its general hypothesis that the interaction between the genome, environmental factors, and hormonal signalling defines mental health states, two cohorts will be investigated within RE-MEND to assess the effects of early environmental exposures, behaviours, and genetics on mental health states during puberty, and to examine whether puberty moderates the relationship between early life stressors and mental illness. The Maternal and Infant Nutrition Interventions, Matlab (MINIMat) cohort from Bangladesh provides detailed information on cognitive function and behavioural data up to age 25, along with data on major life events/trauma, puberty onset, hormones levels, exposure to metals, dietary patterns, and nutritional status. Complementary, the SELMA cohort will provide data from the follow up of the children at 15 years of age, including mental health assessment, behavioural data, puberty onset, and epigenetic data.

To explore more extreme cases of mental health and identify clinical markers of puberty related mental health issues, RE-MEND will establish the clinical cohort (EXIT-fear). This cohort will recruit women aged between 13–17 and 20–25 with anxiety disorders that emerged during puberty. In this clinical study, as well as for a subsample of the SELMA cohort, DNA methylation, brain anatomy, neurochemistry, and neural reactivity will be assessed alongside clinical symptoms and behavioural patterns. Similarly to the early life datasets, genetic information will be converted into and used as polygenic risk scores, and epigenetic information will serve for biomarker discovery as well as within integrated models including mediation of observed outcomes and exposure.

### Peripartum

The peripartum period spans from conception until the end of the first year of the postnatal period. During this time, women undergo major physiological and hormonal changes that can affect their mental health state, making them more prone to mental health issues such as perinatal depression (PND). Globally, 10-20% of women are diagnosed with PND (70), which is classified as a “Major Depression Disorder”. Symptoms range from low mood, anxiety, sleep problems, to more severe symptoms such as suicidal ideation, which accounts for 20% of postpartum deaths (71). The impact of PND does not solely rest with the affected mothers, as children of affected women are at higher risk of behaviour, emotions, and cognitive difficulties later in life (72). Diagnosis of PND is guided by self-reports and manuals such as “Diagnostic and Statistical Manual of mental disorders- fourth and fifth editions” (73), however, reviews have indicated that many guidelines exhibit moderate to low quality, limiting their effectiveness in diagnosis and treatment (74). As a result, many women with PND are often either undiagnosed or untreated, resulting in a significant burden for both the mother and children (75, 76).

Researchers have identified multiple risk factors for PND including hormonal changes within this sensitive period such as those produced by the ovaries (77, 78), but also neurobiological processes involving neurotransmission (79)and dysregulation of the HPA axis function (80, 81), and individual differences in resilience and susceptibility to mental health issues (82). Environmental factors also play a key role in the development of PND during pregnancy. For instance, psychosocial stressors can dysregulate cortisol secretion through HPA axis activation, leading to depressive symptoms (83). Moreover, the drastic fluctuation in the reproductive hormones such as oestrogen and progesterone modulate gamma-aminobutyric acid (GABA) function, lowering stress resilience and heightening susceptibility to depression (84).

Genetic studies have identified several candidate genes found to increase the risk of PND (85), especially those involved in neurotransmitter pathways such *SL6A4* (86), *COMT* and monoamine oxidase (*MAO*) (87), as well as *BDNF* (88, 89). Epigenetic mechanisms further link environmental exposures to genetic risk (90), such as differential methylation of the stress-related gene glucocorticoid receptor gene (*NR3C1*) (91).

Given the large changes in hormonal levels characterising this period, it another life stage to explore RE-MEND’s hypothesis further. To investigate susceptibility and resilience in the perinatal context, two cohorts will be analysed. The Biology, Affect, Stress, Imaging and Cognition cohort (BASIC) (92) provides deep molecular characterization of over 500 women from their first pregnancy visit to six months postpartum, including DNA methylation, transcriptomics, proteomics, metabolomics, and lipidomics. In parallel, the national ongoing mobile application-based study (MoM2B) (93) collects self-reports and digital phenotyping data, such as movement, mobile use, and social media activity of 20,000 pregnancies, with biological samples collected at gestational week 20 and postpartum week 8 for omics analyses. Together, these datasets will enable the identification of molecular markers and predictive models of PND. Here the many different layers of biological information will enable RE-MEND to establish an integrated representation of peripartum depression and identify potential biomarkers. In addition, neuroimaging data from MoM2B participants will be used to profile the neural substrates of PND in terms of brain structure and function.

### Transition into older age

The probability of developing mental health issues advances with age (94), with depression being one of the main risk factors for disability and mortality among the older population (95). This is partly driven by age-related changes in the neuroendocrine system, including declines in both hormonal function and adaptive compensatory mechanisms. Key hormones involved include stress regulation (cortisol), mood and cognitive maintenance (serotonin, norepinephrine, and dopamine), overall well-being and vitality (testosterone and oestradiol), and reproduction (luteinizing hormone (LH), follicle-stimulating hormone (FSH), testosterone, and oestradiol).

In females, the menopausal transition is marked by significant fluctuations in sex hormones, contributing to heightened vulnerability to perimenopausal depression (96). While men experience a slower gradual decline in sex hormones, studies indicated that lower testosterone levels are associated with an increased risk of depression. Testosterone is vital for the proper functioning of the hippocampus, which is a key player in memory, emotion, and mood control. A deficiency in testosterone can therefore contribute to the development and progression of depressive disorders (97). That effect is exemplified by androgen deprivation therapy in men with prostate cancer, which increases the risk of depressive episodes (98).

To address the interaction between hormonal changes and environmental factors, and genetics for mental health states in older ages, RE-MEND will compile and analyse data from Betula, a longitudinal aging cohort study that began 1988 in Umeå, Sweden. Recruitment occurred every five years until 2008–2010 with participants aged 30–80 years at baseline. Each participant was followed up to six times, generating repeated measures of lifestyle, sociodemographic information, genomic, epigenomic, proteomic, and hormonal data. In addition, information regarding the use of specific medications such as anti-depressants prescription and in- or out-patients diagnosis of mental illness will be retrieved from the national archives. These data will be integrated into a single graphical framework to explore the relationships between exposure, covariates, and outcomes, allowing the identification of potential factors that contribute to susceptibility and resilience over the life course.

Findings from Betula will be tested for replication in the MARS (Munich antidepressant response signature) study, a naturalistic open-label longitudinal treatment cohort of inpatients aged 18–80 years. MARS includes repeated weekly assessments of antidepressant treatment outcomes over at least six weeks, enabling the distinction between early and later responders. Validation will occur across multiple molecular layers including genomics, adductomics, epigenomics, metabolomics, proteomics, lipidomics, and hormone levels. For case-control correlations, a healthy subgroup of the Betula study will be age- and sex-matched to MARS participants.

## Experimental studies to validate observations in human data

**Figure 3:**
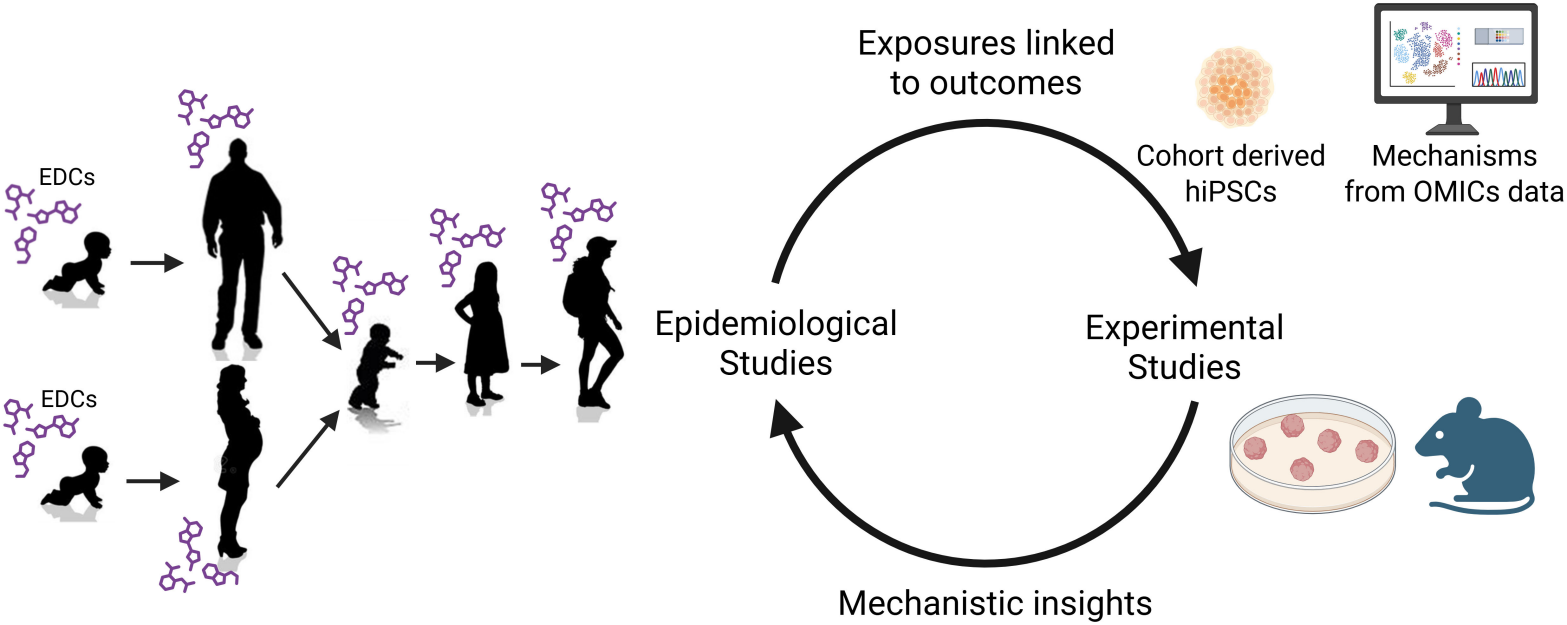
Integration of SELMA and SCDS NC2 cohorts’ findings with *in vivo* and *in vitro* experimental models in RE-MEND. Figure created in https://BioRender.com.

A key component of RE-MEND is to parallel observational studies with experimental models to validate associations within human data and to establish correlative and causal relationships. To model early life events, RE-MEND will employ both *in vitro* and *in vivo* models. The *in vitro* studies will utilize cortical brain organoids derived from induced pluripotent stem cells (iPSCs) obtained from SELMA and SCDS NC2 participants that have been identified as potentially “susceptible” or “resilient” based on biostatistical modelling. In parallel, a mouse study will link the identified environmental factors such as EDC exposure, early life stress (ELS), and nutrition to neuroendocrine and behavioural outcomes (**Figure 3**).

### Cortical brain organoids to perform “epidemiology in a dish”

Differentiation of iPSCs to brain organoid models partly recapitulate salient features of early brain development (99–101). Previous studies have established the importance of endocrine signalling in this process by evaluating transcriptional and morphological disturbances upon exposure to hormone receptor agonists and antagonists (102) as well as an EDC mixture associated with language delay in the SELMA children (103).

In RE-MEND, iPSCs will be generated based on prenatal environmental exposure levels and scored behavioural phenotypes across SELMA and SCDS NC2 participants. More specifically, selection of participants for iPSC generation will be guided by establishing two different categories of particularly vulnerable and resilient individuals, characterised by low exposures and high behavioural phenotypes or high exposures and low phenotypes, respectively. The reprogrammed iPSCs will be differentiated into cortical brain organoid models and exposed to environmental factors identified as protective or harmful from the human cohorts. In this way, the hypothesis that genetic factors drive vulnerability and resilience in these cases will be experimentally addressed and observational data validated. Furthermore, these models will be used for delineating mechanisms linking exposure and functional changes by employing single cell transcriptomics in combination with high-content imaging microscopy screening and electrophysiology of iPSC-derived organoids (104, 105).

### Life stage mouse study to understand mechanisms and effects in a whole organism

In parallel, mouse studies spanning from prenatal neurodevelopment to late age, will enable RE-MEND to explore how risk and protective environmental factors, as well as endocrine challenges, modulate depressive and anxiety-like behaviours across multiple life stages and aid in deciphering, at all stages, the molecular underpinnings of these behaviours.

Systemic hormone imbalances will be explored by administering different concentrations of antagonists targeting relevant hormone receptors. Additionally, interaction with environmental factors, such as EDC exposure or diet, will be explored. To model surgical menopause, ovariectomy will be performed, followed by re-administration of oestradiol and progesterone. Blood and brain tissue will be collected for omics analyses, immune profiling, and hormone measurements, enabling the identification of pathways associated with altered behaviour and providing opportunities for direct comparison with human multi-omics data.

## Developing biomarkers within mental health for diagnosis, disease prevention, and treatment

For clinical assessment, molecular biomarkers in mental health are a key resource that build the foundation for improved future diagnostics.

Generally, biomarkers can be classed into three different groups: 1) Biomarkers of susceptibility, 2) Biomarkers of exposure, and 3) Biomarkers of effect. Biomarkers of susceptibility highlight features that indicate whether an individual may be at risk regardless of their current health state. For example, genetic variants in *SLC6A4* or *BDNF* have been linked to increased susceptibility to depression and anxiety disorders (63, 64, 106). In contrast, biomarkers of exposure define whether an insult has occurred to the system by an external factor which increases the risk of adverse health. Examples include exposure to metals, pesticides and various EDCs that have been shown to increase the risk of depression (107). Finally, biomarkers of effect are the most common type of biomarker in the clinical setting. For example, alteration in the HPA axis function and disbalances in the levels of corticotropic-releasing hormones (CRH), adrenocorticotropic hormone (ACTH), cortisol, mineralocorticoid (MC), and glucocorticoid (GC) are considered hormonal biomarkers of depression (108). In addition, alterations in immune-inflammatory pathways and cytokines levels that affect the normal neuronal function have been linked to mental illness (109). Similarly, metabolites involved in oxidative stress, inflammation, and neurotransmission are considered as biomarkers of anxiety disorder (110).

As many biomarkers reflect dynamic biological processes, particularly biomarkers of effect, timing is critical: it may define whether a minimal treatment regime will be sufficient or whether more intensive therapy is required. Importantly, molecular changes often occur before symptoms appear, making biomarkers a promising tool for early detection of mental health problems, especially in children.

Within RE-MEND, numerous different layers of the molecular machinery are explored to advance mental health research. Key approaches include the use of genomics, epigenomics, transcriptomics, metabolomics, lipidomics (including oxi-lipidomics), proteomics, and a novel unbiased approach to measure chemical modifications of DNA or RNA (adductomics). Metabolomics and lipidomics, in particular, have seen a sharp rise in applications within mental health to evaluate major depressive disorders (111) and anxiety (112). More recently, adductomics has enabled the identification of potential mental health biomarkers through mass spectrometry-based detection of DNA and RNA adducts. Such adducts form when electrophilic chemicals or bioactivated metabolites react with nucleophilic sites on DNA/RNA. In addition, oxidative stress can induce nucleic acid modifications through reactive oxygen species (ROS), leading to lesions such as 8-oxo-7,8-dihydro-2’-deoxyguanosine (8-oxo-dG), and lipid peroxidation induced DNA adducts. While the role of 8-oxo-dG in psychiatric disorders has been implicated (113, 114), the involvement of the broader spectrum of DNA adducts in mental illnesses remains largely unexplored. Using advanced computational approaches, including feature selection strategies and machine learning applied to integrative multi-omics data RE-MEND will aim to create an integrated representation allowing for cross layer biomarker identification within each cohort. While integration across life stages is more complex as the cohorts have significantly different environmental and social backgrounds, RE-MEND will evaluate the knowledge it generates across the whole age range available and how other life stages mediate the identified molecular responses associated to mental health outcomes. This will be further solidified through the experimental work planned within this project.

In essence, RE-MEND aims to utilize various molecular layers to establish novel biomarker panels that can predict susceptibility, resilience, symptoms trajectories, disease state, and treatment response explored across endocrine sensitive life stages. Biomarker validation is a key aspect in the development of such new panels. Within RE-MEND the validation strategy is life-stage specific utilisation of the cohorts. While additional validation outside of RE-MEND is not planned as part of the project, it will be required to validate the biomarkers across a broader range of cultural and social backgrounds. By delineating the relationship between these different OMICs layers, environment and mental health outcomes, RE-MEND will also generate new biological insights and hypotheses about biological pathways involved in mental health states fostering the establishment of personalized treatments and stratification strategies.

## Drug target discovery and repurposing

A high percentage of individuals suffering from mental ill-health remain untreated. Contributing factors include limited availability of mental care facilities, and the adverse effects of drugs given against mental disorders that restrain the patients from adhering to drug intake (115). Moreover, patients’ response to antidepressants and related drugs varies widely, with some developing treatment-resistant conditions, underscoring the urgent need for alternative therapeutic strategies (116, 117). Drug discovery and development are highly dependent on the identification of suitable molecular targets, but the process is time-consuming and costly. To identify new therapeutic uses for existing drugs, RE-MEND is utilizing recent advances in computational drug repurposing that are either data-driven or rational drug repurposing. These approaches offer the potential to accelerate drug development timelines and reduce costs compared to traditional *de novo* drug discovery.

Data-driven drug repurposing, exemplified by the Mode of Action by NeTwoRk Analysis (MANTRA) tool (118), rely on computational analyses of large-scale gene expression datasets to systematically identify drugs whose transcriptional signatures inversely correlate with disease-associated profiles. Specifically, drugs that produce expression profiles opposite to those elicited by a disease, are predicted to reverse or alleviate pathological states and thus become prime candidates for repurposing. Rational drug repurposing, on the other hand, often involves a more target-centric approach. This strategy requires the knowledge of disease-related genes and pathways and then aims at identifying existing drugs that can modulate these targets based on their known mechanisms or their observed effects on gene expression, such as in the Gene2Drug tool (119).

RE-MEND is applying both approaches. In the data-driven drug repurposing, epigenomic and transcriptomic data from the study cohorts is leveraged to identify gene signatures correlated to resilience or susceptibility to mental illnesses, and candidate drugs will be screened for their ability to modulate these signatures. For rational drug repositioning, molecular drug targets will be defined based on genome-scale metabolic modelling, relying on metabolomic markers identified as being associated to mental health outcomes. These targets will be then mapped onto established organ-specific models (liver, kidney, brain) to identify relevant pathways and enzymes as druggable targets. Selection of drugs to be tested will be driven by three factors (1): statistical significance according to the computational analysis (2); well-characterized mechanism of action and (3) potential clinical translatability and safety.

To evaluate whether candidate drugs can cross the blood brain barrier (BBB), a prerequisite for a substance acting on the brain, an *in vitro* human BBB model will be employed. Finally, one to two of the most promising drug candidates will be tested in the mouse model for their impact on molecular endpoints, electrophysiological effects, and behavioural outcomes.

## Increasing mental health literacy is key for developing and communicating effective prevention strategies and reducing stigma

Science communication is widely used to help the public understand complex scientific concepts and to educate interested audiences about the latest research advances and their applications. The effectiveness of science communication depends on the scientists’ ability to understand the audience’s perspectives and their needs, and how their findings can be implemented in healthcare processes. RE-MEND will combine the biological approaches with communication science studies to investigate how the project’s findings can induce changes to medical practices and reduce stigmatization of affected individuals.

To increase the public understanding of how molecular processes and environmental factors contribute to mental health issues, RE-MEND will conduct a survey with representative samples of citizens from different European countries to determine the status of mental health literacy (incl. stigmatization, knowledge, and beliefs about causes and treatment of mental illnesses). Specifically, we will examine as central variables how the emotions of anger and sympathy, as well as the desire for social distance towards people with depression, are expressed in four European countries with different health policies. We will examine the extent to which attributions of responsibility, specifically genetic, environmental, lifestyle, and gene-environment interaction attributions, are related to these central variables in each investigated country. This allows us to learn which attributions of responsibility should be promoted in public and patient health communication in order to reduce the stigmatization of people with depression. To clarify these relationships, we manipulate responsibility cues in online news articles in an experiment conducted in one country and measure their causal relationships between the attribution of responsibility and the central variables. In addition to the general public, health-care professionals are important effectors and contributors to the success of the project’s aims. RE-MEND will conduct qualitative interviews, i.e., semi-structured expert interviews, with psychiatrists as health care professionals to explore their understanding of neurobiological causes and treatments and their attitudes towards neurobiological diagnosis and treatment methods for individuals in the different vulnerable life stages. This provides important evidence-based insights for the development of educational activities, such as workshops and summer schools, for health professionals and scientists in the field as well as recommendations for guidelines development. Following the interviews, communication materials for improved doctor-patient communication will be developed for patients with depression.

RE-MEND will also investigate how individuals at vulnerable life stages (young adults, pregnant women, new parents, menopausal women, and senior citizens) perceive mental health and research findings. This will be achieved through focus groups designed to evaluate the developed communication materials and explore citizens’ understanding of the project outcomes. A citizen science component is focusing on senior citizens. Relevant stakeholders from civil society will first be engaged in focus groups to refine the research gaps and aims. This will be followed by science cafés, which will bring together senior citizens, civil society, and researchers within the field of senior citizens’ health, and collecting narratives about mental health from senior citizens.

The results gained throughout RE-MEND will be translated into recommendations on risk and protective factors, tailored to target groups through prevention, screening, and assessment guidelines. Subsequently, relevant procedures and national and international guidelines will be reviewed and gaps identified based on the new knowledge generated by the project, focusing on the most significant impact and value for end-users.

## Conclusion

RE-MEND aims to bring together uniquely data rich human cohorts and advanced approaches in data-science, biomarker discovery, drug repurposing, neuroimaging, and *in vitro* and *in vivo* experimentation, alongside communication science, to uncover the molecular basis of mental ill-health across the lifespan. The project will deliver validated biomarker panels for susceptibility, disease progression and treatment response; and identify risk and resilience factors and prioritise repurposed drug targets. In parallel, RE-MEND will increase mental-health literacy and reduce stigma, enabling earlier intervention, better management and improved population mental-health.
